# Retratando la Enfermería ariqueña de mediados del siglo XX. Chile, 1950

**DOI:** 10.15649/cuidarte.2709

**Published:** 2023-03-29

**Authors:** Lucia Castillo Lobos, Elizabeth Nuñez Carrasco, Paulina Parrao Cartagena

**Affiliations:** 1 . Escuela de Enfermería Universidad de Santiago de Chile. Santiago. Chile. Email: lucia.castillo@usach.cl Universidad de Santiago de Chile Universidad de Santiago de Chile Chile lucia.castillo@usach.cl; 2 . Escuela de Enfermería Universidad de Santiago de Chile. Santiago. Chile. Email: elizabeth.nunez@usach.cl Universidad de Santiago de Chile Universidad de Santiago de Chile Chile elizabeth.nunez@usach.cl; 3 . Escuela de Enfermería Universidad de Santiago de Chile. Santiago. Chile. Email: pparrao@fonasa.gov.cl Universidad de Santiago de Chile Universidad de Santiago de Chile Chile pparrao@fonasa.gov.cl

**Keywords:** Enfermería, Salud, Historia de la Enfermería, Salud Publica., Nursing, Health, History of Nursing, Public Health., Cuidado, Estudantes, Impacto Psicossocial, Epidemia, Coronavírus.

## Abstract

**Introducción:**

A mediados del siglo XX la ciudad de Arica vivenció cambios socio-políticos que determinaron una precaria situación socio sanitaría. En este tiempo surge la figura de Iris Veliz como primera enfermera profesional de la ciudad y cuyo arribo constituye el punto de partida de la profesionalización de la enfermería del extremo norte de Chile.

**Objetivo:**

Relevar a través de la figura de Iris Veliz la trayectoria de una anónima enfermera chilena sobre quien se ha escrito incipientemente.

**Materiales y Métodos:**

Cualitativo y socio histórico, cuyos hallazgos fueron obtenidos a partir de fuentes documentales secundarias y desde los relatos de quienes conformaron el equipo de trabajo de Iris Veliz en el hospital Juan Noé de Arica sometidos a análisis temático según Flick e interpretados a la luz de los supuestos epistemológicos relativos al reconocimiento femenino de Nancy Fraser.

**Resultados:**

Sobre su figura se destacan las características femeninas como el trato maternal y la virtud, sin embargo, este reconocimiento no alcanza su aporte a la enfermería desde las distintas áreas del rol profesional.

**Discusión:**

Es recordada positivamente, sin embargo, su actuar profesional es invisibilizado por cuanto su campo de acción corresponde a un espacio de prácticas cotidianas y femeninas perpetuándola a un espacio de subyugación producto del ensalzamiento regional de una figura médica dominante.

**Conclusión:**

Iris Veliz es exponente de aquellas profesionales de enfermería que se mantienen ocultas en el espacio restringido de sus acciones y obliga a reflexionar sobre el anonimato de la historia como disruptor de la cultura de los cuidados.

## Abstract (en)

**Introduction:**

In the mid-twentieth century, the city of Arica experienced socio-political changes that led to a precarious social and health situation. During this time, Iris Veliz was the first professional nurse in the city, and her arrival was the starting point for the professionalization of nursing in the extreme north of Chile.

**Objective:**

To highlight through the person of Iris Veliz the trajectory of an anonymous Chilean nurse who has been written about incipiently.

**Materials and methods:**

Qualitative and socio-historical study, its findings emerged from secondary documentary sources and from the accounts of those who were part of Iris Veliz's work team at the Juan Noé Hospital in Arica.

**Results:**

The findings were subjected to Flick's thematic analysis and interpreted in light of Nancy Fraser's epistemological premises regarding women's recognition.

**Discussion:**

She is remembered in a positive way; however, her professional performance is made invisible because her field of action was a space of daily practices traditionally associated with women, which perpetuated her into a state of subjugation as a result of the regional exaltation of a dominant medical figure.

**Conclusions:**

Iris Veliz is an exponent of those nursing professionals who remain hidden in the restricted space of their actions and urges us to reflect on the anonymity of history as a disruptor of the culture of care.

## Resumo

**Introdujo:**

Em meados do século XX, a cidade de Arica experimentou mudanzas sociopolíticas que determinaram uma situagáo sociossanitária precária. Nessa época, surgiu a figura de IrisVeliz como a primeira enfermeira profissional da cidade e cuja chegada constituiu o ponto de partida para a profissionalizagáo da enfermagem no extremo norte do Chile.

**Objetivo:**

Revelar, através da figura de Iris Veliz, a trajetória de uma enfermeira chilena anónima sobre a qual se fez uma escrita incipiente.

**Materiais e Métodos:**

Qualitativos e sócio-históricos, cujos achados foram obtidos a partir de fontes documentais secundárias e das histórias dos integrantes da equipe de trabalho Iris Veliz no hospital Juan Noé de Arica, submetidos a análise temática segundo Flick e interpretados segundo a a luz dos pressupostos epistemológicos relacionados ao reconhecimento feminino de Nancy Fraser.

**Resultados:**

Em sua figura destacam-se características femininas como o tratamento materno e a virtude, porém, esse reconhecimento náo alcanza sua contribuido para a enfermagem a partir das diversas áreas do papel profissional.

**Discussao:**

Ela é lembrada positivamente, porém, suas agóes profissionais sáo invisibilizadas porque seu campo de atuagáo corresponde a um espago de práticas cotidianas e femininas, perpetuando-a em um espago de subjugagáo em decorréncia da exaltagáo regional de uma figura médica dominante.

**Conclusao:**

Iris Veliz é um expoente daqueles profissionais de enfermagem que permanecem ocultos no espago restrito de suas agóes e nos obriga a refletir sobre o anonimato da história como disruptor da cultura do cuidado.

## Introducción

La actual crisis sanitaria por Covid-19 ha significado importantes transformaciones la vida humana que han trascendido a los equipos de salud y en particular a los profesionales de enfermería. Es necesario recalcar que pandemias y momentos de crisis sanitarias han sido parte de los procesos históricos de la humanidad y por ello se ha despertado el interés por estudiar sobre los cimientos históricos de la enfermería generando producción científica en torno al tema.

En este afán surge desde Latinoamérica la figura de Iris Veliz Hume; enfermera chilena, ariqueña, quien en medio de la pampa chilena supo dedicar su vida a combatir la mortalidad infantil a mediados del siglo XX. Si bien, el fenómeno presentado corresponde a un acontecimiento local situado en Chile, la magnitud del fenómeno en el periodo de tiempo referido alcanzó ribetes inconmensurables en la población infantil chilena que fue diezmada por enfermedades como tifus y tuberculosis, encumbrando a Chile dentro del grupo de países con mayor tasa de mortalidad infantil de la época^1^.

El propósito de este artículo es invitar a analizar, desde un enfoque socio histórico, el despliegue de las prácticas de enfermería de mediados del siglo XX desde la voz de sus propios protagonistas construyendo un vínculo con los orígenes de la profesión. El objetivo es relevar, a través de la figura de Iris Veliz, la trayectoria de una anónima pero destacada enfermera chilena sobre quien se ha escrito incipientemente. Es así como surgen las siguientes preguntas de investigación ¿Cómo fue el cotidiano de las prácticas del cuidado profesional frente a la mortalidad infantil en Arica de mediados del siglo XX? ¿Cómo ejerció las áreas del rol de enfermería en este escenario de crisis? ¿Por qué su aporte al desarrollo de la ciencia de enfermería ha sido invisibilizado?

Según lo expuesto, desde los supuestos teóricos relativos al reconocimiento femenino^2^, su recuerdo nos acercará a suprimir aquellas prácticas reproductoras de olvido y ocultamiento de la profesión contribuyendo a fortalecer los cimientos históricos de la enfermería conformando un corpus histórico epistemológico de mujeres^3^ latinoamericanas como manifestación de “nuestra realidad y de nuestras experiencias.”^4^ Hoy es el turno de Iris Veliz; quien ha esperado pacientemente su reconocimiento hasta este momento.

## Materiales y Métodos

Investigación cualitativa con enfoque histórico social a partir de la revisión de fuentes secundarias relativas a textos salud de mediados del siglo XX y de fuentes primarias orales aportadas por relatos de vida de informantes claves. El grupo de estudio fue conformado por 11 octogenarias mujeres chilenas, seleccionadas a través de muestreo teórico, las cuales compartieron el atributo de conocer de manera personal a Iris Véliz además de conformar su equipo de trabajo en el Servicio de Pediatría del Hospital Juan Noé de Arica desde 1953. La técnica recolección de datos correspondió a entrevista semiestructurada y focus group en enero del año 2018 previa firma de consentimiento informado. La teorización de estos relatos en categorías de análisis fue almacenada en Mendeley Data17.^5^

Los hallazgos celosamente atesorados fueron sometidos a análisis temático del discurso según la propuesta metodológica de Flick^6^, según categorías definidas y emergentes relativas al rol profesional asistencial, docente, gestor y de investigación de enfermería^7^ por cuanto aúnan un conjunto de prácticas propias del cuerpo de conocimiento y estructura disciplinar configurando una identidad profesional de enfermería^8^. Posteriormente, los hallazgos obtenidos fueron sometidos a triangulación de investigadores para asegurar la validez, coherencia y complemento del análisis desde una perspectiva teórica^9^.

Desde un marco interpretativo identitario y de género inspirado en los supuestos teóricos del reconocimiento que, según Fraser, centra las injusticias enraizadas en los patrones sociales de representación, interpretación y comunicación^2^, se espera rescatar aquellas prácticas de enfermería situadas en un momento histórico particular, y a través de la transformación social, cultural y simbólica de estas comunicaciones, se intenta contribuir al reconocimiento de las identidades individuales de enfermería desde el interior de la propia comunidad enfermera y con ello, alcanzar el reconocimiento social de la profesión^10^.

Esta investigación está enmarcada en la línea de investigación histórica de la Escuela de Enfermería de la Universidad de Santiago de Chile y ha sido aprobada por el Comité de Ética Institucional. Dicyt Regular 021902CL Memorias de la Enfermería Chilena: un siglo al cuidado de personas, familias y comunidades. 

## Resultados

Chile es un país ubicado en el extremo sur de Latinoamérica caracterizado por una larga extensión de territorio dividida en 16 regiones que comprende un extenso desierto, valles y lagos. Iris Veliz desarrolló su vida personal y trayectoria profesional en la ciudad de Arica en el extremo norte de Chile y frontera con Perú, ubicada a 2.0037 kms de la ciudad de Santiago, capital económica y política de Chile.

*“La situación en Arica no era muy boyante, porque era gente de trabajo, y mucha gente no tenía un trabajo estable, porque aquí no existía mayormente la parte laboral, aquí existían los valles, Azapa y Yuta y la mayoría de las personas trabaja en la parte de cultivo”. (Informante N°7).*

Durante el gobierno de Carlos Ibáñez del Campo la ciudad de Arica inicia una etapa de crecimiento económico centrado en el desarrollo del régimen de franquicias tributarias, zona franca y áreas destinadas al asentamiento industrial que permitieron invertir en el mejoramiento de la ciudad en obras viales, equipamiento público y aumento de las viviendas luego de adquirir su condición de “puerto libre” ^11^.

*“...Arica, que no tenía ningún atractivo, pasó a ser un atractivo enorme porque era un sitio del país en que podía importar lo que viniera sin pagar estos tremendos impuestos. Se transforma en puerto libre muy atractivo y empieza a llegar mucha gente..." (Informante N°10)*

El crecimiento de la ciudad trajo consigo un aumento de la población, fenómeno explicado por la migración transfronteriza cuantificada en un 5% del total de extranjeros para la región^12^ y por la migración rural-urbana más alta del país que alcanzó un 92%12. Simultáneamente, el país comienza a experimentar un crecimiento poblacional alcanzando 5.023.539 (1940)12, 5.932.995 (1950^12)^y 7.374.115 (1960) habitantes^12^. Para 1960 la expectativa de vida había mejorado exponencialmente desde 31 a 54,4 años para los hombres y de 32 a 59,9 años para las mujeres^13^.

La institucionalidad sanitaria de la ciudad de Arica fue representada por el Hospital San Juan de Dios el cual fue reconstruido y rebautizado en 1952 con el nombre de Dr. Juan Noé Crevani^14^ en honor a un destacado médico italiano cuyo aporte a la salud pública nacional recae en la erradicación de la malaria endémica en el norte de Chile en 1945^15^, sumándose a este reconocimiento la denominación de otras laureadas instituciones regionales. Si bien la construcción del hospital Dr. Juan Noé se constituye como emblema del desarrollo económico de la región, los aumentos de sus capacidades fueron insuficientes para albergar la sobre exigencia asistencial debido al carácter de puerto libre que adquiere la región.

*“...los impuestos que se recaudaron fueron invertidos ahí en esa zona y un hospital que era pequeño, antiguo, se transforma en un hospital moderno para veinticinco mil personas. Pero cuando se entrega el hospital, el año 52, Arica ya no tenía veinticinco mil personas, subió de once mil, trece mil, se fue a cuarenta mil. Por lo tanto, desde el punto de vista sanitario todo se quedó chico y todo en Arica se quedó realmente chico y empezó a llegar mucha gente de Santiago, Valparaíso, Concepción." (Informante N°10)*

La modernidad ofrecida por la construcción del recién inaugurado Hospital Dr. Juan Noé Crevani, no trasciende a la atención de sus pacientes por cuanto la enfermería era sustentada en el oficio y carácter religioso situación que dista de la concentración profesional de enfermeras en la capital^16^. Esta distribución inequitativa de la enfermería nacional obligó a delegar acciones de enfermería a monjas, mayordomos y practicantes, en una suerte de dualidad en la administración de la salud a lo largo del país producto del aislamiento geográfico.

*"...no había personal de auxiliares (de enfermería). Había como cuatro o cinco practicantes y se iban preparando a través de la práctica que hacían con los enfermos y quienes los guiaban eran los médicos porque no había otras personas.porque acá no llegaba ni el tren. Para llegar acá tenían que llegar en avión o por el mar que eran las únicas entradas en ese tiempo en 1953”. (Informante N°7).*

*"...la parte administrativa del servicio estaba en manos del contador y la parte de entrega de medicamentos y ropa a cargo de las monjitas que hicieron un trabajo bastante bueno para ese tiempo, no tanto en la parte técnica pero sí en comportamiento”. (Informante N°7).*

La creación del Servicio Nacional de Salud en el año 1952 ^17^, significó la fusión de todas las instituciones de salud existentes en la época y la consolidación de la destinación de enfermeras profesionales a todo el país en un afán descentralizador de la nueva institucionalidad sanitaria chilena. Así, en 1953 ingresaron las tres primeras enfermeras profesionales al Hospital Juan Noé Crevani y en este espacio historiográfico Iris Veliz hace arribo a la ciudad de Arica casi 50 años después del inicio de la profesionalización universitaria de las enfermeras chilenas. Posteriormente en 1955, el recién fundado Servicio Nacional de Salud creó dos nuevos cargos de enfermeras en Arica; una como Enfermera Jefa del Centro de Salud y otro como Enfermera Jefa del Hospital Dr. Juan Noé Crevani^18^.

### I. La Enfermera: Iris Veliz

Iris Veliz Hume nació el 20 de mayo de 1927 en la ciudad de Arica, en el extremo norte de Chile, en el seno de una familia de clase media. Hija de padre carpintero y madre dueña de casa, quienes trabajaban arduamente entregando “pensión” a quienes visitaban la región (Informante N°11). Estudió enfermería en la Universidad de Chile y decide volver a su ciudad natal para ejercer su profesión^19^. Fue la primera enfermera profesional en ejercer en la ciudad de Arica y conformó el primer grupo de enfermeras universitarias llegadas a la ciudad en conjunto a Filomena Garrido y Josefina Morales en 1953^20^^,^^21^.

*"La primera enfermera que llegó a Arica se llamó Iris Veliz, que llegó el 15 de marzo de 1953. Quince días después llegó la Josefina Morales con la Filomena Garrido, entonces entre las tres hicieron el primer equipo que tuvieron a cargo todo el hospital” (Informante N°2)*

**Enfermera Iris Veliz Hume en sus primeros años de ejercicio profesional:**
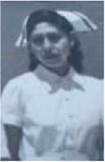
*Fuente: Archivos familiares-Hospital Juan Noé de Arica.*

La obra de Iris Veliz alcanza protagonismo con la creación del primer Servicio de Pediatría de la Provincia de Arica en el Hospital Dr. Juan Noé Crevani inaugurado en 1952, lugar donde ejerció por más de 20 años. Durante este periodo de su vida se casó y tuvo dos hijos. Falleció el 13 de marzo de 1976 en pleno ejercicio de la profesión^21^. En reconocimiento a su trayectoria uno de los principales Centros de Salud Familiar de la ciudad de Arica fue bautizado con su nombre, no obstante, su denominación ha sufrido variados cambios arbitrarios en una suerte de invisibilización desde la institucionalidad y que solo ha sido posible recuperar a través de iniciativas familiares.

Quienes la conocieron la describen físicamente como una mujer que destacaba por su estampa, que no pasaba desapercibida; “la Sra. Iris me impresionaba, ¿será el porte? (Informante N°4). Además de su indistinguible figura, en ella se ensalzan valores como la abnegación, entrega profesional y humanización de su cuidado debido a la impronta “maternal” de su trato al “trabajar incansablemente y sin horario”^22^.

*“...Ella como Ariqueña de origen, conoció muy bien la gente, el modo de ser, la idiosincrasia del nortino ariqueño y ella tenía una llegada bastante fácil y grata con los padres de los niños y era muy maternal en su trato..." (Informante N°10).*

La llegada de Iris Veliz se convierte en punto de inflexión en la atención de salud ariqueña marcada por la precariedad de los cuidados de enfermería, la alta demanda asistencial y la miseria de los espacios de subsistencia de sus pequeños pacientes.

*“...para Iris Veliz fue un desafío para hacerse cargo como enfermera: había muchos más niños que atender, había cosas mucho más complejas que hacer y ese fue el desafío que asumió..." (Informante N°10)*

Desde este momento se instaurarán, por primera vez, prácticas de enfermería profesional materializados en cuidados profesionales de mayor complejidad que hasta la fecha se habían mantenido vedados debido a la ausencia de enfermeras profesionales, impidiendo el tratamiento eficaz de los niños dada la imposibilidad de contar con elementos terapéuticos como plasmoterapia, transfusiones y fleboclisis^22^.

*“.ahí se necesitaban no sólo los médicos, sino una enfermera que se quedara a cargo del niño en cosas tan complejas como los niños quemados o un extra sanguíneo por el factor RH. De tal manera, que había un verdadero equipo de gente que le dio un nivel mucho más alto a este servicio de pediatría" (Informante N°10).*

Simultáneamente, estadísticas de la época dan cuenta que en 1956 “fallecieron 100 niños menores de un año por diarrea que en su mayoría poseía distrofia y de ellos un 90% no poseían control de salud eficiente”^23^.

*“Las enfermedades prevalentes que había en ese tiempo era la diarrea. En noviembre- diciembre ya comenzábamos a realizar las campañas de prevención de diarrea: el lavado de manos, es en lo que más insistimos “(Informante N°2)*

*“Hubo harta polio en ese tiempo también y los casos graves de dificultad respiratoria se trasladaban a Antofagasta, que era lo más cercano.". “Era una época difícil, fallecían mucho." (Informante N°2)*

*“En esos años llegaron muchos niños con tuberculosis al servicio de pediatría y también habían niños con meningitis" (Informante N°6)*

A partir de estos relatos vienen a la mente imágenes de salas de hospital atiborradas de niños enfermos y malnutridos alejados de sus madres. Fue a este grupo más frágil al que Iris dedicó tenazmente un rol maternal y protector de los derechos de infancia muchas veces vulnerados por la propia ignorancia de sus padres e invisibilizados por la sociedad en las que les tocó vivir.

*“Había una gran problemática con los enfermos menores de un año: llegaban todos desnutridos, La enfermedad de los niños no era una neumonía, sino que una diarrea, mal alimentados por sus mamás que no tenían grandes conocimientos del cuidado de sus niños, y no tenían un niño, sino que tres, cuatro o cinco niños y todos chiquitos”. (Informante N°7) “...los niños morían como moscas, morían tres o cuatro niños diarios y llegaban en pésimas condiciones (Informante N°7).*

Iris Veliz debió sortear la impronta de la pobreza y el escaso desarrollo tecnológico y sanitario de la región para el cuidado de sus pequeños pacientes, sin embargo, la precariedad material fue compensada con amor y dedicación prodigado en cada una de sus acciones de cuidado.

*“Yo sé que en realidad habían muy pocas camas. En una cama estaban unos (niños) para arriba y otros para abajo y los niños no solamente podían estar uno arriba y otro abajo, sino que podía haber más porque eran niños que tenían ocho o diez meses o un año que parecían niños de tres meses. Eran niños desnutridos extremos” (Informante N°7).*

*“Las incubadoras eran las cajas de zapatos” (Informante N°7).*

Desde el rol gestor de enfermería el liderazgo y habilidades de Iris Veliz permitieron instaurar procesos clínicos fundamentados en el conocimiento disciplinar adquirido durante sus estudios en la Universidad de Chile. Así, Iris Veliz logró establecer un sistema profesional de trabajo de manera progresiva y respetuosa dando cuenta del dominio de un importante capital relacional.

*“con mucha paciencia ellas (las enfermeras) empezaron a organizarse y a tomar el papel que les correspondía como enfermeras. Fueron muy cautelosas, no llegaron a atropellarnos ni tirarnos de acá para allá, sino que dejaron a las monjitas que las ayudaran con la ropa” (Informante N°2)*

Respecto a la gestión clínica puso en marcha la estandarización de registros clínicos y sistematización de la atención de enfermería. *“Ella organizó el servicio de una manera ya diferente, con un sentimiento más moderno para la época porque se trabajaba sin mayores elementos. Se abrieron registros para el tratamiento de los niños, entregas de turnos, recibos de turno y todas las novedades que pasaban durante las 24 hrs”...” todo estaba normado” (Informante N°10).*

La inédita implementación de prácticas profesionales relacionadas con la elaboración de registros clínicos^24^ permitió asegurar la continuidad terapéutica y disminuir la incidencia de errores con ocasión de la atención de los niños y niñas ariqueños. Iris Veliz fue además fue una excelente gestora de recursos físicos y materiales ya que “nunca hubo restricción para los niños, el servicio estuvo muy bien abastecido y muy bien dirigido” (Informante N°10).

*“..el gran aporte de ella fue dotar al servicio de pediatría de todo un sistema y de unas disposiciones sanitarias para que los tratamientos médicos, se cumplieran. No sólo la parte del tratamiento médico, si no en todos los aspectos del servicio, pero del servicio tenía que preocuparse de que todo estuviera bien aseado, el cambio de las ropas, los tratamientos efectuados y que los niños fueran bien recibidos y se fueran en buenas condiciones (Informante N°10)*

Dentro de sus principales desafíos profesionales, Iris Veliz asumió la misión de formar al personal técnico de enfermería a su cargo motivando permanente la superación del personal de su dependencia trasladando incluso el cuidado maternal entregado a sus pequeños pacientes al espacio de las relaciones establecidas con el personal a su cargo.

*“La Sra. Iris me consiguió una beca para estudiar, hice el curso (de técnico paramédico) becada. Después cuando terminó el curso ella me puso la toca y me llevó a pediatría" ...’’Yo la quise mucho, conmigo fue una mujer que me enseñó a ser mujer, profesional y emocionalmente”. (Informante N° 9)*

*“.además de jefa fue mamá, fue preocupada hasta de la parte personal de cada una de nuestras compañeras" (Informante N°8)*

Finalmente, desde el rol profesional de investigación y pese a la existencia de investigaciones realizadas durante el tiempo en que Iris Veliz ejerció en el Hospital Juan Noé de Arica^23^, no se encuentran antecedentes que den cuenta de su participación en trabajos de esta naturaleza.

Como contrapunto a las sentidas expresiones sobre la memoria de Iris Veliz, existen relatos que, si bien reconocen su trayectoria, la restringen a un estricto espacio doméstico de sus acciones impidiendo que su figura trascienda a las amplias esferas de reconocimiento de la salud pública nacional.

*“En realidad, es sorprendente que sea un CESFAM el que lleve su nombre, porque realmente Iris nunca trabajó fuera del hospital, nunca atendió en un policlínico periférico, cómo había otras enfermeras que sí lo hicieron. Ella realmente estuvo concentrada en el servicio de pediatría. parecía mucho más lógico que una sal o la neonatología llevara el nombre de Iris, dentro del hospital porque su trabajo fue intrahospitalario" (Informante N10)*

**Enfermera Iris Veliz Hume (1975):**
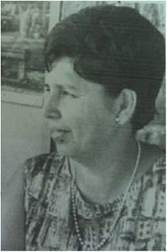
*Fuente: Archivos familiares-1975*

Desde este hecho surge la siguiente reflexión; ¿Por qué se posterga la figura de Iris Veliz pese a que su nombre está en el ideario de la comunidad ariqueña? ¿Por qué su recuerdo se reduce a los límites de quienes la conocieron?

## Discusión

La trayectoria de Iris Veliz como enfermera pionera en el extremo norte de Chile a mediados del siglo XX implica variadas transformaciones de la enfermería ariqueña por cuanto su llegada constituye un tránsito desde la enfermería de oficio hacia la enfermería profesional Ariqueña. Esto revela el retraso de la enfermería regional en que los cuidados eran homologables a un oficio doméstico^25^ ejercidos por mujeres de “baja extracción social” y religiosas, dotadas sólo del conocimiento adquirido a través de la historia oral de la tradición. Iris Veliz es innovadora y pionera en la instauración de un sistema de cuidados en virtud de su formación universitaria a través de la aplicación de un cuerpo de conocimiento profesional propio^8^ según los últimos estándares sanitarios de enfermería de la época en un escenario sociosanitario adverso agravado por el desfase existente entre la producción de conocimiento entre la capital y el resto del país.

El relevo de Iris Veliz permite deconstruir antiguas prácticas de enfermería que integran las distintas áreas del rol profesional cuando esta definición aún se encontraba en la reflexión de las intelectualidades nacionales^26^. Desde el rol asistencial los relatos hacen gala de su desenvolvimiento en la atención de sus pacientes y familias. Como gestora fue excepcionalmente innovadora para la época. Como educadora, Iris Veliz trabajó con ahínco y exigencia fusionados en un liderazgo comparable al amor que prodigan las madres a sus hijos erigiéndola en una suerte de gran madre universal. Estas características asociadas a la identidad femenina y a las bases disciplinares en que “la enfermera es el espejo en el que se reflejaba la situación de la mujer a través de los tiempos” ^27^ pero que a su vez son invisibilizados al supeditarse a valores femeninos propios del cuidado y de la enfermería, y por lo tanto subalternos y subyugados, por cuanto el acto de cuidar constituye un acto primitivo^28^.

Desde el rol de investigación se avizora un vacío dada la inexistencia de hallazgos que den cuenta de su desempeño en esta área. Se infiere que esta brecha en el área científica estaría perfilada por la exclusiva dedicación de Iris Veliz al cuidado directo de los niños enfermos por sobre las acciones reflexivas e intelectuales en torno al cuidado que desestimaron su formación profesional para actividades investigativas. Este alejamiento de las actividades académicas se condice con la evolución de la disciplina a nivel latinoamericano en que el desarrollo científico y teorización ha tomado fuerza recién desde mediados del siglo XX encontrándose aún en ciernes^29^.

Los relatos dan cuenta de una sobrevaloración de acciones de enfermería vinculados a la disposición del ámbito clínico para el ejercicio protagónico del rol médico tales como; saneamiento, cumplimiento de órdenes y aseo por cuanto integran funciones homologables al trabajo doméstico impuestas en un régimen militarizado y de subordinación. El exhibir virtudes como veracidad, maternidad, caridad, limpieza, observación, puntualidad y discreción^30^ constituyen cualidades vinculadas a la identidad femenina propias de toda enfermera virtuosa y exitosa para la época y sugieren que, para organizar y reorganizar el sistema profesional de trabajo de enfermería, Iris Veliz debió desplegar estrategias de liderazgo situacional a través de un lenguaje disciplinar, pero sumiso.

Iris Veliz ha sido postergada y su aporte a la salud pública ha sido adornado por permanentes adulaciones que excluyen su verdadero valor profesional. Su figura ha sido restringida al espacio particular de sus acciones, ocultada bajo el manto hegemónico de la imagen médica como curador de la salud y en donde la enfermería se concibe bajo su yugo paternalista^28^ sin trascender al espacio médico y hegemónico de poder.

La desestimación de Iris Veliz es la antítesis de la sobrevaloración de otros hitos sanitarios regionales vinculados a la práctica médica y que han acaparado todo el reconocimiento de la salud pública perpetuando la histórica subyugación de la enfermería como consecuencia de una práctica social de menosprecio de lo femenino. Esta desestimación alcanza al gremio de enfermería en que las luces de las enfermeras académicas e intelectuales chilenas de mediados del siglo XX^25^ opacaron la figura de otras enfermeras pioneras y anónimas. Iris Veliz no pertenecía a este selecto grupo social de enfermeras y tampoco dedicó sus esfuerzos a actividades científicas. Ella dedicó su vida al cuidado de la infancia indefensa y carente en medio de la pampa chilena.

El desconocimiento generalizado del aporte de Iris Veliz a la enfermería chilena se acrecienta con el paso del tiempo y la perpetúa en las sombras ¿Será su no pertenencia a la élite intelectual? ¿Será su dedicación al desempeño asistencial? ¿Será la connotación femenina de las prácticas de cuidado? ¿Será la ubicación social de sus sujetos de cuidado?

## Conclusiones

El analizar la obra de Iris Veliz da cuenta sobre una trayectoria que tuvo como impronta la abnegación, entrega y humanización de los cuidados. No obstante, es una fiel representante del tortuoso camino que la enfermería ha debido recorrer para posicionarse como profesión independizada de la figura médica y masculina como extensión de los quehaceres de la realidad doméstica y femenina.

Su aporte radica en ser pionera en la implementación de prácticas profesionales de enfermería en el extremo norte de Chile que permitió que la vulnerable infancia Ariqueña recibiese cuidados de una mano maternal, pero, por sobre todo profesional. Del mismo modo, esta configuración de un quehacer considerado doméstico, es una acción de implementación que significo mejoras de la atención de salud de la infancia Ariqueña con la consecuente mejora de sus indicadores de salud.

Este hito constituye la piedra angular para su reconocimiento desde el interior de la comunidad de enfermería, en una suerte de vínculo identitario con el pasado que permite a la figura de Iris Veliz trascender a un lugar de reconocimiento de la misma magnitud de otros renombrados próceres de la salud pública regional y nacional. Su trayectoria ejemplifica el mérito de los ejecutores de las políticas públicas al mismo nivel de las intelectualidades en salud.

La figura de Iris Veliz corporiza a tantas otras enfermeras anónimas y opacadas por el dominio masculino y elitista e invita a reflexionar sobre la infravaloración de la profesión de enfermería en contrapunto con la sobrevaloración de la hegemonía intelectual médica. Estas líneas servirán de desagravio para que, a través del reconocimiento de la trayectoria individual de una mujer- enfermera por parte la comunidad de enfermería, contribuir al reconocimiento del grupo social de enfermería por parte de la comunidad general, como un acto político de reivindicación y reconocimiento profesional femenino.

Iris Veliz inspira, transporta e invita a imaginar. A ella todo nuestro reconocimiento.
